# Intravenous fosphenytoin therapy for the rescue of acute trigeminal neuralgia crisis in pre- and post-neurosurgical patients: a retrospective observational study

**DOI:** 10.3389/fneur.2024.1493274

**Published:** 2025-01-07

**Authors:** Shusaku Noro, Hideki Endo, Bunsho Asayama, Yuki Amano, Masahiro Okuma, Ryota Nomura, Kaori Honjo, Yoshinobu Seo, Hirohiko Nakamura

**Affiliations:** Department of Neurosurgery, Nakamura Memorial Hospital, Sapporo, Japan

**Keywords:** trigeminal neuralgia, acute exacerbation, fosphenytoin, phenytoin, numerical rating scale, microvascular decompression

## Abstract

**Background:**

There is no established treatment for the acute exacerbation of trigeminal neuralgia. We aimed to investigate the efficacy and safety of intravenous fosphenytoin for this disease.

**Methods:**

We conducted a retrospective observational study of data from 41 patients with trigeminal neuralgia who received intravenous fosphenytoin therapy. Fosphenytoin diluted with physiological saline was administered intravenously at a loading dose of 9.8–20.7 mg/kg or at a dose of 7.5–9.5 mg/kg when maintenance therapy was needed. Pain was evaluated using a numerical rating scale (NRS), assessed immediately before administration (baseline) and at 2, 12, and 24 h after administration.

**Results:**

The mean (± standard deviation) NRS score was 9.85 ± 0.69, 0.49 ± 1.47, 1.60 ± 2.19, and 3.46 ± 3.19 at baseline, 2, 12, and 24 h after administration, respectively (*p* < 0.001). Intravenous fosphenytoin therapy was effective for the acute exacerbation of trigeminal neuralgia regardless of whether it was administered during the perioperative period of microvascular decompression (MVD) or the type of drugs used concomitantly. Fosphenytoin was effective when re-administered (*n* = 14) or at a maintenance dose (*n* = 2). The adverse drug reactions observed were mild dizziness in six patients, abnormal auditory perception and thirst in three patients each, and somnolence, decreased SpO_2_, and drug eruption in one patient each, all of which were transient.

**Conclusions:**

Intravenous fosphenytoin therapy can immediately eliminate pain during acute exacerbation of trigeminal neuralgia and can be a useful therapeutic drug in emergency response or until elective treatment, such as MVD, is performed.

## 1 Introduction

Trigeminal neuralgia is induced when a trigger zone is stimulated by activities of daily living, such as eating, tooth brushing, face washing, and talking, and is characterized by sudden unilateral pain attacks along the branches of the trigeminal nerve (1). The International Classification of Headache Disorders (3rd Edition) classifies trigeminal neuralgia into three categories. Typically, trigeminal neuralgia is associated with the compression of the trigeminal nerve by blood vessels. Secondary trigeminal neuralgia is caused by compression by a tumor or demyelination of the nerve (e.g., multiple sclerosis or brain infarction). The causes of idiopathic trigeminal neuralgia cannot be determined by examinations, such as electrophysiological tests and magnetic resonance imaging (2, 3). Various antiepileptics and other drugs are recommended for typical trigeminal neuralgia and idiopathic trigeminal neuralgia (primary trigeminal neuralgia) based on the results of a systematic review and other studies. For secondary trigeminal neuralgia, pharmacotherapy should be similar to that for primary trigeminal neuralgia, although evidence regarding this is sparse. Thus, currently, pharmacotherapy does not differ based on the type of trigeminal neuralgia (2).

Treatment guidelines (2, 4–6) recommend carbamazepine or oxcarbazepine as the first-line pharmacotherapy for trigeminal neuralgia, and carbamazepine is widely used in Japan. Baclofen and lamotrigine are recommended for second-line pharmacotherapy, although these agents are used off-label. Despite appropriate treatment with any of these pharmacotherapeutic strategies alone or in combination, intractable pain due to the acute exacerbation of trigeminal neuralgia develops in some patients. Occasionally, persistent pain attacks lasting for days or weeks occur during severe episodes. Microvascular decompression (MVD) and trigeminal nerve blocks are effective. However, performing these procedures in emergency medical care settings is challenging. These procedures are often performed electively, and the waiting time for MVD at our hospital is 5–30 days.

Oral agents are not suitable for pain relief during acute exacerbations. Japanese treatment guidelines recommend the following drugs as effective fast-acting analgesics, although the strength of the recommendation is low because no randomized controlled trials have been conducted (1).

- Local anesthetics such as lidocaine (administration route: eye instillation, nasal or oral route, injection into the trigger point, intravenous infusion, or nerve block) are used.
- Anticonvulsants such as phenytoin or fosphenytoin (intravenous infusion) are used.
- Serotonin agonists such as sumatriptan (subcutaneous injection or nasal route) are used.

Fosphenytoin is an anticonvulsant commonly used to treat status epilepticus. Fosphenytoin, a prodrug of phenytoin, is used to treat adverse reactions associated with phenytoin, such as injection site pain and phlebitis. It is rapidly converted in the plasma to its active form, phenytoin, in equivalent (equimolar) amounts and can be safely administered intravenously. Phenytoin blocks Na^+^ channels by suppressing the influx of Na^+^, after which depolarization of neurons and neurotransmission within axons are less likely to occur. Through this mechanism, phenytoin specifically suppresses only the abnormal frequency of discharge or “firing” of nerves and does not suppress normal, less frequent discharge activity of nerves. Carbamazepine is the first-line drug for the treatment of trigeminal neuralgia; therefore, voltage-dependent Na^+^ channels are considered to be involved in the pathology of trigeminal neuralgia, and reports indicate that the firing of nerve potential is a cause of pain in trigeminal neuralgia (7, 8). Voltage-dependent Na^+^ channels are thought to be involved in the mechanism of action of carbamazepine and fosphenytoin. The use of fosphenytoin for the acute exacerbation of trigeminal neuralgia has already been reported (9–11).

We have also used fosphenytoin off-label out of necessity for patients with acute exacerbation of trigeminal neuralgia for whom effective procedures such as MVD could not be performed in the emergency response and have previously reported some cases (12). Since then, we have accumulated more experience and obtained new findings, reported herein. In this study, we aimed to investigate the efficacy and safety of intravenous fosphenytoin as a therapy for the acute exacerbation of trigeminal neuralgia.

## 2 Methods

### 2.1 Study population

We included data from 41 patients who were diagnosed with typical trigeminal neuralgia and received intravenous fosphenytoin between September 2015 and November 2023 at Nakamura Memorial Hospital (Sapporo, Japan). We included patients with trigeminal neuralgia who were aged ≥ 18 years, of any sex, experiencing acute exacerbations, regardless of prior medication, MVD, nerve blocks, or gamma knife surgery. Patients with a history of allergy to fosphenytoin and those with serious arrhythmias, including sinus bradycardia and heart block, were excluded. The following facts were explained to the patients: there are risks of adverse drug reactions caused by fosphenytoin, such as dizziness, gait disturbance, nystagmus, dysarthria, ataxia, diplopia, sleepiness, palpitations, phlebitis, hypotension, and arrhythmia; the use of fosphenytoin is off label; and no other procedures are available for emergencies. The patients accepted these risks. Consent for participation in this study was obtained from all the patients. The study protocol was approved by the Ethics Committee of Nakamura Memorial Hospital (Approval No. 2024090201) and conformed to the Declaration of Helsinki and its later amendments.

### 2.2 Administration method

Fosphenytoin was diluted with physiological saline (0.9% NaCl) and administered intravenously using an infusion pump (50 mg/min). The loading dose was 9.8–20.7 mg/kg (750–1,200 mg/dose), and the maintenance dose was 7.5–9.5 mg/kg (422–750 mg/dose). Patients receiving fosphenytoin were observed by nurses, underwent electrocardiography, and received non-invasive blood pressure monitoring. They were instructed to rest in bed for 1 h after the procedure. The fosphenytoin used was Fostoin (750 mg), which is commercially available in Japan (Nobelpharma Co., Ltd., Tokyo, Japan).

### 2.3 Endpoints

Facial pain was evaluated using a numerical rating scale (NRS) to determine efficacy. The NRS is an 11-point scale of pain severity, ranging from 0 to 10, with 0 indicating no pain and 10 indicating the most severe pain experienced by the patient. Patients evaluated their pain. The NRS score was evaluated immediately before administration (baseline) and at 2, 12, and 24 h after administration.

The degree and frequency of adverse drug reactions that developed after intravenous fosphenytoin therapy, including dizziness, gait disturbance, nystagmus, dysarthria, ataxia, diplopia, sleepiness, palpitations, phlebitis, hypotension, and arrhythmia, were evaluated for safety. Moreover, clinical laboratory test data related to hepatic and renal functions, including creatinine, urea nitrogen, liver enzymes, and gamma-glutamyl transferase, were evaluated.

### 2.4 Statistical analyses

The NRS scores immediately before administration (baseline) and at each time point (2, 12, and 24 h) after administration were compared using a paired *t-*test. A *p*-value of < 0.05 was considered statistically significant. The R software (version 4.4.0) was used for statistical analyses.

## 3 Results

Data from all 41 patients were included in this study and no data were excluded (clinical data of patients treated for trigeminal neuralgia crisis in Supplementary Table 1). Table 1 presents the summary of patient characteristics and treatment history. All patients were diagnosed with typical trigeminal neuralgia. The patients' mean age was 63.2 years (range, 23–88 years); 58.5% were female.

**Table 1:** Summary of patient characteristics and treatment history.

		***N***	**41**
Sex	Male	17	(41%)
	Female	24	(59%)
Age (years)	Mean ± SD	63.2 ± 16.4
	Median (min, max)	68 (23, 88)
Weight (kg)	Mean ± SD	58.3 ± 11.0
	Median (min, max)	55.2 (36.2, 79.0)
TN classification	Classical trigeminal neuralgia	41	(100%)
TN laterality	R	23	(56%)
	L	18	(44%)
TN distribution	V1	1	(2%)
	V2	19	(46%)
	V3	6	(15%)
	More than 1 branch	15	(37%)
Concomitant medication^*^	Na^+^ channel blocker	31	(76%)
	Ca^2+^ channel α2δ ligand	19	(46%)
	Na^+^ channel blocker and Ca^2+^ channel α2δ ligand	9	(22%)
	Two or more drugs	12	(29%)
	None	0	(0%)
Treatment history for TN (therapy)^*^	MVD	10	(24%)
	Nerve block	8	(20%)
	γ-knife	2	(5%)

Ca, calcium; L, light; Max, maximum; Min, minimum; MVD, microvascular decompression; Na, sodium; R, right; SD, standard deviation; TN, trigeminal neuralgia.

^*^Duplicates present.

Intravenous fosphenytoin was administered 62 times in 41 patients. Ten of the 41 patients (24%) received it on two different occasions, and four patients (10%) received it on three or more different occasions (maximum: six occasions). Among the 62 courses of intravenous fosphenytoin therapy, 25 were administered to inpatients, 37 to outpatients, 11–10 outpatients who were hospitalized immediately (24%), and 16–9 emergency outpatients (22%).

**Figure 1:**
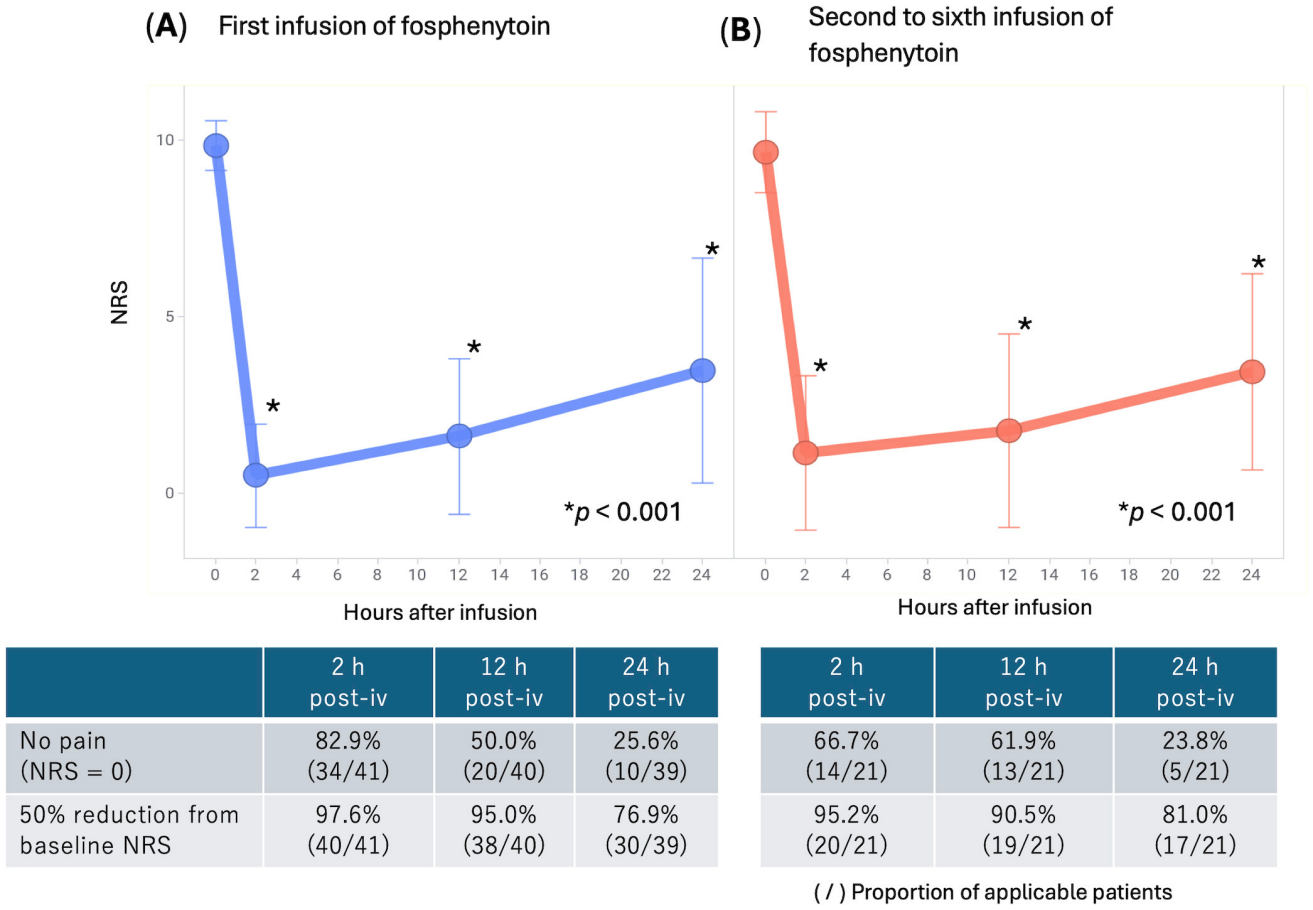
Changes in NRS scores after intravenous infusion of fosphenytoin. **(A)** Average NRS scores and standard deviation are shown for all patients (*n* = 41) who received the first infusion of fosphenytoin. **(B)** Average NRS scores and standard deviation are shown for patients (*n* = 14, 21 infusions in total) who received fosphenytoin infusion several times (except the first infusion). NRS, numerical rating scale.

Changes in NRS scores after fosphenytoin administration are depicted in Figure 1A. At baseline, the NRS score was, on average (±standard deviation), 9.85 ± 0.69 (range, 6–10). The NRS score was 0.49 ± 1.47 2 h after administration (*p* < 0.001), 1.60 ± 2.19 12 h after administration (*p* < 0.001), and 3.46 ± 3.19 24 h after administration (*p* < 0.001). Thus, NRS scores significantly decreased at all time points after administration compared to before administration, and the inhibitory effect on pain lasted for 24 h or longer after administration. The proportion of patients whose NRS score decreased by ≥50% compared to at baseline was 97.6% 2 h after administration and 76.9% 24 h after administration. In 82.9% of the patients, pain disappeared completely (NRS score = 0) 2 h after administration.

After the initial administration, 14 patients (a cumulative total of 21 patients) received intravenous fosphenytoin therapy multiple times on different occasions (Figure 1B). When fosphenytoin was administered multiple times (two or more), the NRS score was 9.67 ± 1.15 at baseline, 1.14 ± 2.20 (*p* < 0.001) 2 h after administration, 1.76 ± 2.76 (*p* < 0.001) 12 h after administration, and 3.42 ± 2.79 (*p* < 0.001) 24 h after administration. Thus, as with the initial administration, the NRS score significantly decreased at any time point after administration compared to baseline, and the inhibitory effect on pain lasted for 24 h or longer after administration. The proportion of patients whose NRS scores decreased by ≥50% compared to the baseline was 95.2% 2 h after administration and 81.0% 24 h after administration.

**Figure 2:**
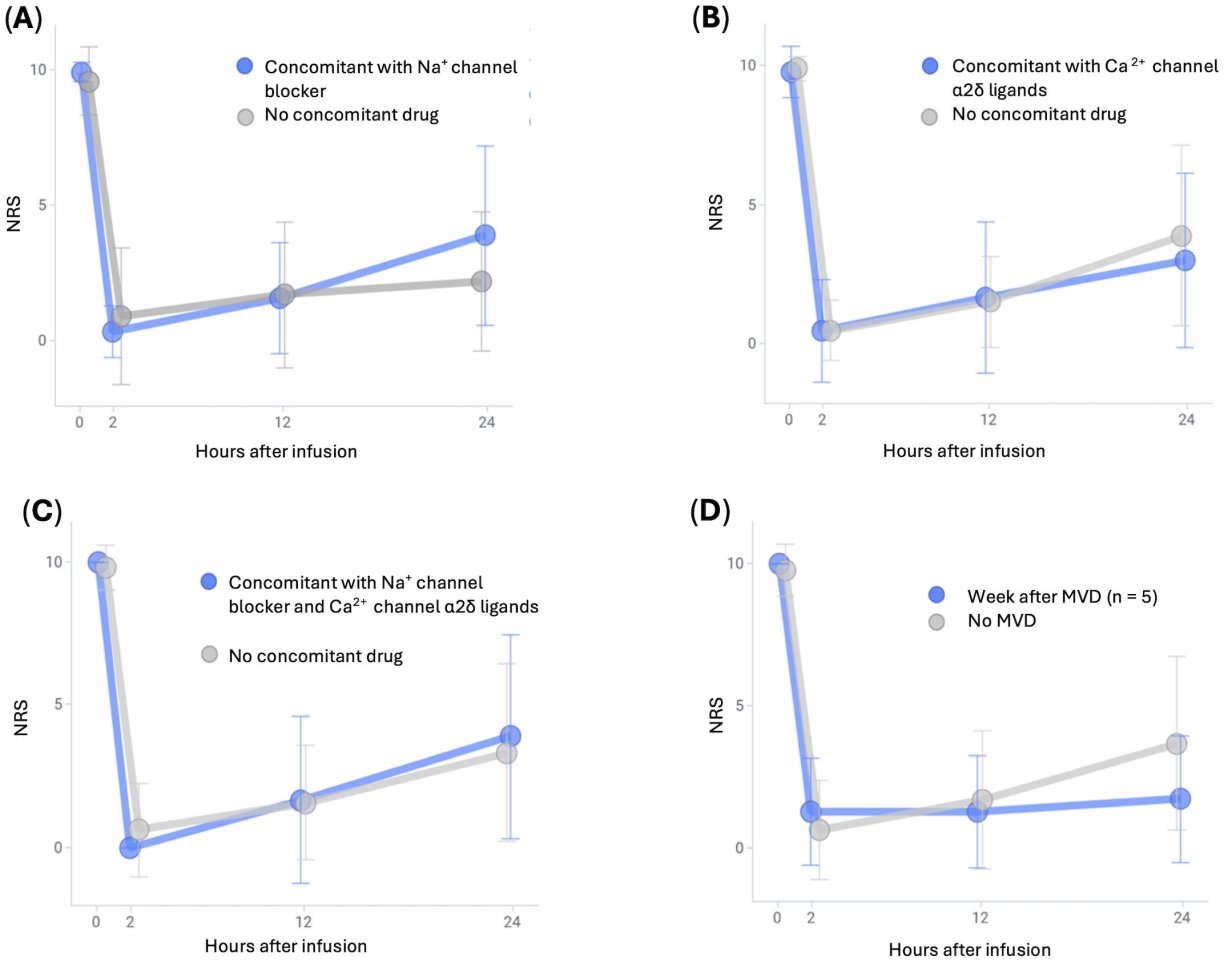
Changes in NRS scores after intravenous infusion of fosphenytoin in patients stratified by their background. **(A)** Average NRS scores and standard deviation for patients with or without oral Na^+^ channel blockers. **(B)** Average NRS scores and standard deviation for patients with or without oral Ca^2+^ channel α2δ ligands. **(C)** Average NRS scores and standard deviation for patients with or without oral Na^+^ channel blockers and Ca^2+^ channel α2δ ligands. **(D)** Average NRS scores and standard deviation for patients with or without MVD on the preceding day. MVD, microvascular decompression; NRS, numerical rating scale.

Changes in NRS scores after fosphenytoin administration according to the type of oral drug used to suppress trigeminal neuralgia crisis are shown in Figures 2A–C. Regardless of the type of oral drug used, intravenous fosphenytoin therapy had a similar effect on the acute exacerbation of trigeminal neuralgia. When intravenous fosphenytoin therapy was administered to seven patients in whom immediate pain relief was not achieved soon after MVD (within 1 week), pain relief was observed in all seven patients, similar to other patients (Figure 2D).

**Figure 3:**
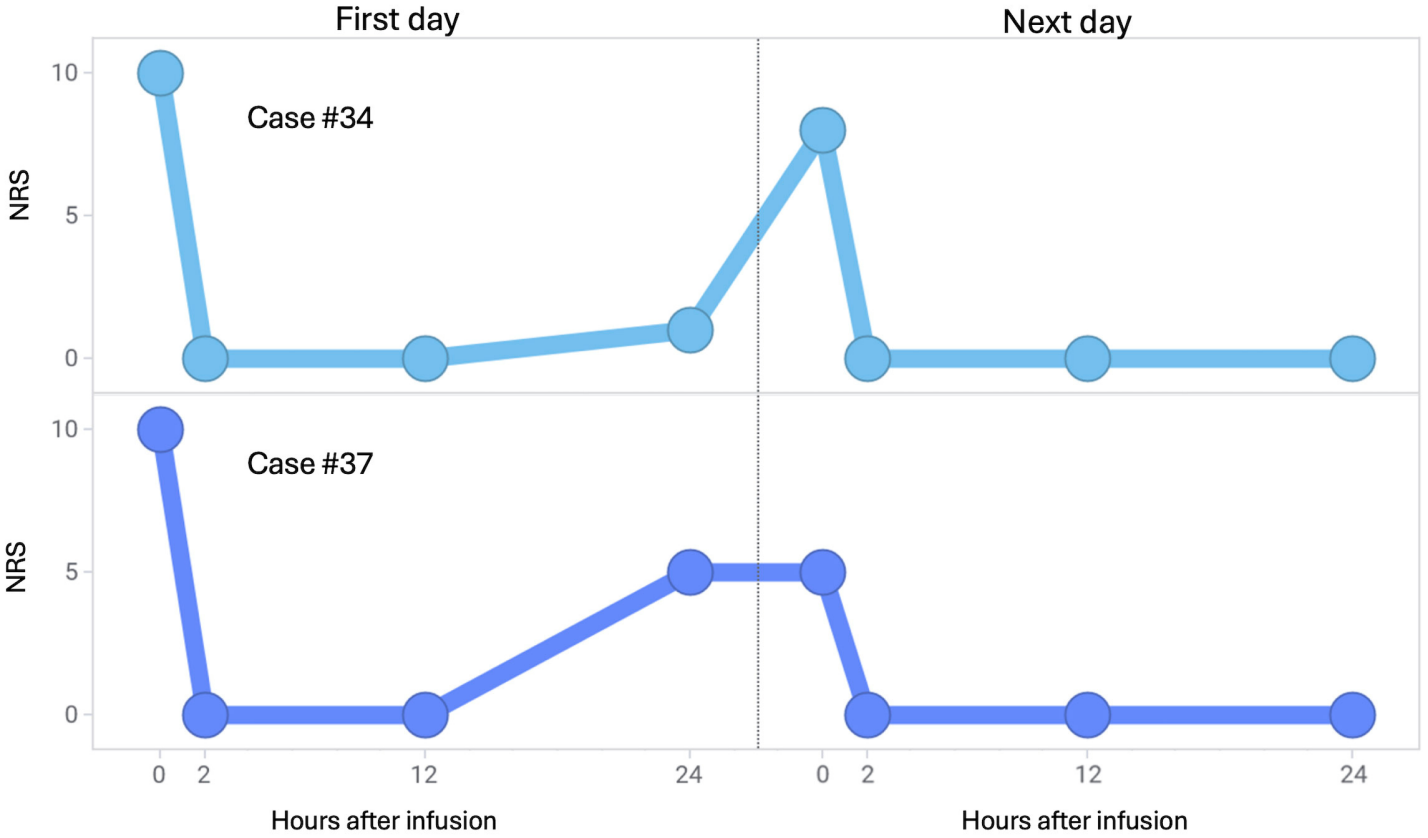
Changes in NRS scores after intravenous infusion of fosphenytoin for individual patients. (Upper panel) Case 34 was a 69-year-old woman who received fosphenytoin at a dose of 14.4 mg/kg on the first day and at the same dose on the next day. (Lower panel) Case 37 was a 50-year-old man who received fosphenytoin at a dose of 15.2 mg/kg on the first day and at 9.5 mg/kg on the next day. NRS, numerical rating scale.

Changes in the NRS scores of two patients who received maintenance therapy because their NRS scores increased on the day after the initial dose was administered are depicted in Figure 3. After maintenance therapy, the patients' NRS scores decreased again.

The adverse drug reactions observed were mild dizziness in six patients, abnormal auditory perception and thirst in three patients each, somnolence, decreased SpO_2_, and drug eruption in one patient each, all of which were transient. All the patients recovered within the same day without treatment. None of the patients experienced nystagmus or dysarthria as an adverse drug reaction. The patient who developed a drug eruption was allergic to several drugs. The patient received an intravenous injection of hydrocortisone sodium succinate and was cured within several hours of treatment. No adverse drug reactions that raised concerns were observed, and fosphenytoin was well-tolerated.

## 4 Discussion

The present study demonstrates the efficacy and safety of administering intravenous fosphenytoin therapy in reducing pain due to trigeminal neuralgia crisis in 41 patients. Regardless of whether it was administered during the perioperative period of MVD and the type of drugs used concomitantly, the inhibitory effect on pain lasted for 24 h or longer after administration. This therapy is effective for treating trigeminal neuralgia crisis on different occasions in the same patient. The findings of this study will contribute to the development of treatments for trigeminal neuralgia.

The pain associated with trigeminal neuralgia is considered one of the most intense types of pain. Pain is usually controlled by administering oral drugs, such as carbamazepine. However, some activities of daily living trigger sudden pain attacks. During attacks, patients may crouch with their hands on their faces and become unable to talk, eat alone, or drink. It is not uncommon for them to be rushed to the hospital. However, hospitals have no established or simple treatment. If a nerve block or emergency surgery, such as MVD, cannot be performed, there are no further options.

At our hospital, we have attempted to treat patients seeking emergency care with intravenous injections of the anticonvulsant fosphenytoin since 2015. For the 41 patients whose data have been accumulated to date, the NRS scores after fosphenytoin administration were significantly lower than the baseline NRS scores until at least 24 h after administration. The proportion of patients whose NRS score at 2 h after administration decreased by 50% or more compared to that at baseline (initial administration) was 97.6%, and the proportion of patients whose pain disappeared completely (NRS score = 0) was 82.9%, indicating that pain relief with this drug is effective and rapid. This effect persisted for 24 h or longer after administration.

Although few papers address managing acute TN crises, a systematic review and guidelines summarize the current evidence. They suggest that lidocaine can be administered via nasal spray, local nerve block, or intravenous infusion. There is some evidence supporting the use of botulinum toxin and a recommendation for subcutaneous sumatriptan to alleviate acute TN attacks. Intravenous lidocaine, phenytoin, and fosphenytoin are also proposed for inpatient treatment (13).

Andersen et al. (14) reported prospective observations of 15 patients who received intravenous fosphenytoin during the exacerbation of trigeminal neuralgia, and 60% (nine) of responders experienced a 50% decrease in pain intensity 24 h after administration. In line with these observations, our results are consistent with these reports, confirming that intravenous fosphenytoin therapy is an effective treatment for acute exacerbations.

Schnell et al. (15) investigated the intravenous administration of phenytoin and reported that it was administered to 18 patients with trigeminal neuralgia in an emergency room on 65 occasions and that immediate pain relief was observed in 89.2% of the cases. In contrast, our study indicates that IFT provides a superior and safer alternative for immediate TN crisis relief, with only mild adverse events such as dizziness, thirst, and abnormal auditory perception, which were transient, and the drug was well-tolerated and safe.

Interestingly, when fosphenytoin was administered soon after MVD (within 1 week) to seven patients in whom immediate pain relief was not achieved following MVD, pain disappeared in four patients, and the NRS score decreased from 10 at baseline to 2 in two patients, and from 10 at baseline to 5 in one patient 2 h after administration, indicating that the anxiety experienced by the patients who still had pain after surgery was successfully eliminated. Generally, the time to pain relief after MVD varies from individual to individual; it may be achieved immediately after surgery but may also take several weeks. The demonstration of the effect of this drug on pain relief after surgery indicates that the drug provides a measure to address remaining pain after surgery, which we consider encouraging for physicians.

In all patients whose data were included in this study, trigeminal neuralgia was managed by oral drugs such as Na^+^ channel blockers or Ca^2+^ channel α2δ ligands, and the effect of fosphenytoin was not affected by the type of oral drug. Similar to carbamazepine, fosphenytoin is a Na^+^ channel blocker. Therefore, acute exacerbation of trigeminal neuralgia in patients taking Na^+^ channel blockers may have resulted in resistance. However, the results of this study refute this possibility and are clinically valuable in that fosphenytoin can be used without the need to ask patients about the type of oral drugs used for pain management.

Although fosphenytoin is clinically positioned as a therapeutic drug for acute exacerbation of trigeminal neuralgia, it cannot be expected that this drug will continue to be effective for 24 h or longer after administration. However, in the present study, when two patients received maintenance therapy because the pain relapsed on the day after this drug was initially administered, the maintenance therapy suppressed pain as well as the initial administration.

The mechanism by which fosphenytoin alleviates trigeminal neuralgia pain remains to be fully elucidated. Previous studies have suggested that cerebral blood flow (CBF) can be modulated by neurogenic and metabolic mechanisms (16, 17). Interestingly, phenytoin has demonstrated a protective hemodynamic effect on CBF (18), whereas a decrease in CBF has been observed during trigeminal ganglion stimulation, which mimics nerve hyperexcitability during paroxysmal attacks (19). These findings raise the possibility that changes in CBF may play a role in the mechanism by which fosphenytoin alleviates pain, potentially counteracting the hemodynamic effects of trigeminal nerve hyperactivity. Further investigations are warranted to confirm this hypothesis.

The present study has the following limitations, and the results must be interpreted cautiously. First, this was a retrospective study conducted at a single center with a single administration group. Therefore, bias may have been introduced in the results. Second, the loading and maintenance doses of this drug were set within the range of anticonvulsant doses, and pharmacokinetic investigations were not performed. Therefore, this issue could not be considered or discussed.

The results described above suggest that intravenous fosphenytoin therapy immediately eliminates pain during acute attacks of trigeminal neuralgia and that fosphenytoin can be a useful therapeutic drug in emergency response or until elective treatment, such as MVD, is performed. Therefore, verification of fosphenytoin in prospective studies is warranted.

## 5 Summary

This retrospective observational study investigated the efficacy and safety of intravenous fosphenytoin in the management of acute exacerbations of trigeminal neuralgia in pre- and post-neurosurgical patients. The study analyzed data from 41 patients, and pain was evaluated using a numerical rating scale (NRS). Fosphenytoin was diluted in physiological saline and administered intravenously. The mean NRS score was 9.85 before administration, 0.49 after 2 h, 1.60 after 12 h, and 3.46 after 24 h (*p* < 0.001 for all), suggesting that treatment with intravenous fosphenytoin can rapidly eliminate pain during acute trigeminal neuralgia crisis. Intravenous fosphenytoin therapy was also beneficial regardless of prior treatment, including microvascular decompression, nerve blocks, or gamma knife surgery, and was effective when administered on multiple occasions or as maintenance therapy. The adverse reactions were generally mild and transient and included dizziness, abnormal auditory perception, and somnolence. These findings suggest that intravenous fosphenytoin is a valuable emergency treatment option for trigeminal neuralgia, offering immediate pain relief and serving as a bridge to elective treatment. Further prospective studies are recommended to validate these results. This study aligns with *Frontiers in Neurology*'s focus on innovative therapeutic approaches for neurological conditions.

## Data Availability

The original contributions presented in the study are included in the article/Supplementary material, further inquiries can be directed to the corresponding author.
