# Supplementary material for: Intravenous fosphenytoin therapy for the rescue of acute trigeminal neuralgia crisis in pre- and post-neurosurgical patients: a retrospective observational study
Source: Front Neurol. 2025 Jan 7;15:1493274. doi: 10.3389/fneur.2024.1493274 (PMC11746063; doi:10.3389/fneur.2024.1493274)
Supplement: Supplementary file 1 [file Table_1.docx]

Supplementary Material

# Supplementary Table 1. Clinical data of patients treated for trigeminal neuralgia crisis

| Patient | Times | Age | Sex | Side | Branch | Weight  (kg) | Dose of fPHT | | NRS | | | | MVD  (days from dosing) | Concomitant drug | ADR | In/outpatient |
| --- | --- | --- | --- | --- | --- | --- | --- | --- | --- | --- | --- | --- | --- | --- | --- | --- |
|  |  |  |  |  |  |  | mg | mg/kg | Immediately before | 2 h | 12 h | 24 h |  |  |  |  |
| 1 | ➀ | 77 | M | L | V2 | 55.0 | 750 | 13.6 | 10 | 3 | 2 | 2 | – | CBZ 400 mg | – | Outpatient |
| 2 | ➀ | 41 | F | R | V3 | 50.0 | 750 | 15.0 | 10 | 0 | 2 | 5 | – | CBZ 200 mg | – | Outpatient |
| 3 | ➀ | 82 | M | L | V2V3 | 55.0 | 750 | 13.6 | 10 | 0 | 5 | 5 | – | CBZ 200 mg  PGB 50 mg* | – | Outpatient |
| 4 | ➀ | 78 | F | L | V2V3 | 52.7 | 750 | 14.2 | 10 | 0 | 0 | 2 | – | PGB 275 mg | – | Emergency outpatient |
|  | ➁ | 78 | F | L | V2V3 | 52.7 | 750 | 14.2 | 10 | 0 | 0 | 2 | – | PGB 275 mg | – | Emergency outpatient |
|  | ➂ | 78 | F | L | V2V3 | 52.7 | 750 | 14.2 | 10 | 0 | 0 | 2 | 12 | PGB 275 mg | – | Emergency outpatient |
| 5 | ➀ | 83 | M | L | V1V2 | 72.0 | 750 | 10.4 | 10 | 0 | 2 | 2 | 27 | PGB 300 mg | – | Inpatient |
| 6 | ➀ | 53 | F | L | V3 | 46.9 | 750 | 16.0 | 10 | 0 | 0 | 6 | – | CBZ 800 mg | – | Outpatient |
|  | ➁ | 53 | F | L | V 2V 3 | 52.7 | 750 | 14.2 | 10 | 0 | 0 | 5 | 3 | CBZ 800 mg | – | Outpatient |
| 7 | ➀ | 59 | F | R | V 2 | 65.8 | 750 | 11.4 | 10 | 0 | 4 | 7 | – | CBZ 600 mg | – | Outpatient |
|  | ➁ | 59 | F | R | V 2 | 65.8 | 750 | 11.4 | 10 | 1 | 5 | 8 | 11 | CBZ 600 mg  TRAM+AcA1T* | – | Emergency outpatient |
| 8 | ➀ | 74 | M | L | V 2 | 72.3 | 750 | 10.4 | 10 | 0 | 5 | 8 | 18 | CBZ 400 mg  PGB 150 mg  PGB 75 mg* | – | Outpatient |
| 9 | ➀ | 62 | M | L | V 3 | 76.8 | 750 | 9.8 | 10 | 0 | 0 | 5 | 4 y before  23 | CBZ 600 mg | – | Outpatient |
|  | ➁ | 62 | M | L | V 3 | 76.8 | 750 | 9.8 | 10 | 0 | 5 | 6 | 1 day before | CBZ 400 mg  PGB 350 mg | – | Inpatient |
| 10 | ➀ | 63 | M | L | V 2 | 57.0 | 750 | 13.2 | 10 | 0 | 3 | – | – | CBZ 300 mg  PGB 75 mg* | – | Emergency hospitalization |
| 11 | ➀ | 82 | M | R | V 2 | 53.0 | 750 | 14.2 | 10 | 0 | 0 | 2 | 55 | CBZ 400 mg  PGB 150 mg | Dizziness | Emergency hospitalization |
| 12 | ➀ | 80 | F | R | V 1V 2 | 50.0 | 750 | 15.0 | 10 | 2 | 0 | 0 | 7 days before | CBZ 300 mg | Dizziness | Inpatient |
|  | ➁ | 80 | F | R | V 1V 2 | 50.0 | 750 | 15.0 | 10 | 0 | 0 | 4 | 1 y before | CBZ 200 mg  PGB 100 mg | – | Emergency hospitalization |
| 13 | ➀ | 68 | M | R | V 2V 3 | 72.6 | 750 | 10.3 | 10 | 0 | 0 | 5 | 41 | CBZ 600 mg  PGB 450 mg  TRAM+AcA4T | – | Outpatient |
|  | ➁ | 68 | M | R | V 2V 3 | 72.6 | 750 | 10.3 | 10 | 0 | 0 | 5 | 20 | CBZ 600 mg  PGB 450 mg  TRAM+AcA4T | – | Outpatient |
| 14 | ➀ | 60 | F | R | V 3 | 71.0 | 750 | 10.6 | 10 | 0 | 0 | 0 | 6 y before  27 | PGB 450 mg  LCM 100 mg* | – | Outpatient |
| 15 | ➀ | 82 | F | R | V 1V 2 | 50.0 | 750 | 15.0 | 10 | 1 | 1 | 1 | 14 | PGB 150 mg  TRAM+AcA3T  LCM 50 mg* | Dizziness | Emergency hospitalization |
| 16 | ➀ | 78 | F | L | V 2V 3 | 65.8 | 750 | 11.4 | 10 | 0 | 0 | 3 |  | PGB 150 mg  TRAM+AcA3T | – | Outpatient |
| 17 | ➀ | 86 | F | L | V 2 | 44.4 | 750 | 16.9 | 10 | 0 | 2 | 3 | 299 | CBZ 800 mg  TRAM+AcA4T | Dizziness | Emergency hospitalization |
| 18 | ➀ | 68 | F | R | V 2 | 55.2 | 750 | 13.6 | 10 | 0 | 1 | 1 | – | LCM 200 mg | – | Emergency hospitalization |
|  | ➁ | 68 | F | R | V 2 | 55.2 | 750 | 13.6 | 10 | 0 | 0 | 2 | 6 | LCM 200 mg | – | Emergency hospitalization |
|  | ➂ | 68 | F | R | V 2 | 55.2 | 750 | 13.6 | 10 | 5 | 1 | 1 | 1 day before | LCM 200 mg | – | Inpatient |
| 19 | ➀ | 80 | F | L | V 2V 3 | 59.0 | 750 | 12.7 | 10 | 0 | 1 | 1 | 8 | PGB 200 mg  PGB 50 mg* | – | Inpatient |
| 20 | ➀ | 69 | M | R | V 2 | 69.0 | 750 | 10.9 | 10 | 0 | 0 | 3 | 69 | LCM 400 mg  PGB 150 mg  ZNS 200 mg | – | Outpatient |
|  | ➁ | 69 | M | R | V 2 | 69.0 | 750 | 10.9 | 10 | 2 | 0 | 2 | 2 days before | LCM 400 mg  PGB 150 mg  ZNS 200 mg | – | Inpatient |
|  | ➂ | 69 | M | R | V 2 | 69.0 | 750 | 10.9 | 10 | 0 | 0 | 5 | – | LCM 400 mg  PGB 150 mg  ZNS 200 mg | – | Emergency hospitalization |
| 21 | ➀ | 69 | F | R | V 2V 3 | 45.7 | 750 | 16.4 | 10 | 0 | 3 | 5 | 4 y before  3 y before | LCM 200 mg  LCM 50 mg* | – | Emergency hospitalization |
| 22 | ➀ | 36 | M | L | V 1V 2 | 65.0 | 750 | 11.5 | 10 | 0 | 2 | 6 | – | LCM 200 mg  PGB 150 mg | – | Emergency outpatient |
|  | ➁ | 36 | M | L | V 1V 2 | 65.0 | 750 | 11.5 | 10 | 0 | 2 | 5 | 5 | LCM 200 mg  PGB 150 mg | – | Emergency outpatient |
| 23 | ➀ | 71 | M | R | V 1V 2 | 68.0 | 750 | 11.0 | 10 | 0 | 8 | 10 | 10 | CBZ 600 mg  GBP 600 mg  PGB 150 mg | – | Emergency outpatient |
| 24 | ➀ | 88 | M | R | V 2 | 71.0 | 750 | 10.6 | 10 | 0 | 0 | 0 | 12 | CBZ 600 mg  PGB 75 mg | – | Emergency outpatient |
| 25 | ➀ | 46 | F | L | V 2 | 46.0 | 750 | 16.3 | 10 | 1 | 1 | 3 | – | CBZ 600 mg | – | Emergency outpatient |
|  | ➁ | 46 | F | L | V 2 | 46.0 | 750 | 16.3 | 10 | 2 | 3 | 4 | 42 | CBZ 600 mg | – | Outpatient |
| 26 | ➀ | 23 | F | L | V 2 | 50.0 | 750 | 15.0 | 10 | 0 | – | – | – | CBZ 200 mg | – | Emergency outpatient |
| 27 | ➀ | 43 | F | R | V 2V 3 | 46.0 | 750 | 16.3 | 10 | 0 | 5 | 5 | – | PGB 150 mg | – | Emergency outpatient |
| 28 | ➀ | 32 | M | L | V 3 | 75.0 | 750 | 10.0 | 10 | 0 | 3 | 3 | 1 day before | LCM 300 mg | Drug eruption | Inpatient |
| 29 | ➀ | 43 | F | R | V 2 | 50.0 | 750 | 15.0 | 10 | 1 | 2 | 8 | 7 | CBZ 300 mg | – | Emergency hospitalization |
| 30 | ➀ | 58 | F | R | V 2V 3 | 51.5 | 750 | 14.6 | 10 | 0 | 0 | 0 | 73 | CBZ 350 mg  PGB 75 mg* | – | Outpatient |
| 31 | ➀ | 75 | F | R | V 2 | 41.6 | 750 | 18.0 | 10 | 0 | 0 | 0 | 2 y before  1 day before | CBZ 200 mg  PGB 75 mg* | – | Inpatient |
| 32 | ➀ | 69 | M | L | V3 | 67.0 | 750 | 11.2 | 10 | 0 | 0 | 0 | 3 y before  1 y before  1 day before | LCM 300 mg | – | Inpatient |
| 33 | ➀ | 67 | F | R | V1 | 62.0 | 750 | 12.1 | 10 | 0 | 0 | 0 | 17 | MGB 30 mg  LCM 50 mg* | – | Emergency hospitalization |
| 34 | ➀ | 69 | F | R | V 2 | 52.0 | 750 | 14.4 | 10 | 4 | 4 | 10 | – | CBZ 600 mg | Thirst, auditory disorder | Outpatient |
|  | ➁ | 69 | F | R | V 2 | 52.0 | 750 | 14.4 | 10 | 5 | 5 | 5 | 9 | CBZ 600 mg  PGB 75 mg* | Thirst, auditory disorder | Outpatient |
| 35 | ➀ | 68 | M | R | V1V2V3 | 69.4 | 1200 | 17.3 | 10 | 0 | 0 | 0 | 7 | CBZ 800 mg  MGB 20 mg | Dizziness | Inpatient |
| 36 | ➀ | 64 | F | R | V2 | 56.2 | 1012 | 18.0 | 10 | 0 | 0 | 1 | 12 y before  5 y before | CBZ 800 mg  MGB 10 mg | Dizziness, somnolence oxygen saturation decreased | Inpatient |
|  | ➁ | 64 | F | R | V2 | 56.2 | 421.5 | 7.5 | 8 | 0 | 0 | 0 | – | CBZ 800 mg  MGB 10 mg | – | Inpatient |
| 37 | ➀ | 50 | M | L | V1V2V3 | 79.0 | 1200 | 15.2 | 10 | 0 | 0 | 5 | – | CBZ 800 mg | Thirst | Inpatient |
|  | ➁ | 50 | M | L | V1V2V3 | 79.0 | 750 | 9.5 | 5 | 0 | 0 | 0 | 40 | CBZ 800 mg | – | Inpatient |
| 38 | ➀ | 64 | M | R | V2 | 67.1 | 1200 | 17.9 | 8 | 0 | 0 | 0 | – | CBZ 200 mg | Thirst, auditory disorder | Outpatient |
| 39 | ➀ | 46 | F | R | V2 | 49.0 | 750 | 15.3 | 10 | 8 | 8 | 8 | 8 | PGB 50 mg | – | Outpatient |
| 40 | ➀ | 45 | F | L | V2 | 50.0 | 900 | 18.0 | 6 | 0 | 0 | 0 | – | PGB 50 mg | Auditory disorder | Outpatient |
| 41 | ➀ | 40 | F | R | V2 | 36.2 | 750 | 20.7 | 10 | 0 | 0 | 10 | – | CBZ 200 mg | – | Outpatient |
|  | ➁ | 40 | F | R | V2 | 36.2 | 750 | 20.7 | 10 | 0 | 0 | 0 | – | CBZ 200 mg | – | Emergency outpatient |
|  | ➂ | 40 | F | R | V2 | 36.2 | 750 | 20.7 | 10 | 0 | 0 | 0 | – | CBZ 200 mg  PGB 150 mg | – | Emergency outpatient |
|  | ➃ | 41 | F | R | V2 | 36.2 | 750 | 20.7 | 10 | 8 | 8 | 8 | – | CBZ 200 mg  PGB 150 mg | – | Emergency outpatient |
|  | ➄ | 41 | F | R | V2 | 36.2 | 750 | 20.7 | 10 | 1 | 8 | 8 | – | CBZ 200 mg  PGB 150 mg | – | Emergency outpatient |
|  | ➅ | 41 | F | R | V2 | 36.2 | 750 | 20.7 | 10 | 0 | 0 | 0 | – | CBZ 200 mg  PGB 150 mg | – | Emergency outpatient |

ADR, adverse drug reaction; CBZ, carbamazepine; F, female; fPHT, fosphenytoin; GBP, gabapentin; IFT, intravenous fosphenytoin therapy; L, left; LCM, lacosamide; M, male; MGB, mirogabalin; MVD, microvascular decompression; NRS, numerical rating scale; PGB, pregabalin; R, right; TRAM+AcA, tramadol hydrochloride + acetaminophen; ZNS, zonisamide.

*Oral drugs added 12–24 hours after IFT
